# Dupilumab-Induced Vitiligo Managed Effectively With Nemolizumab and Targeted Topical Therapy

**DOI:** 10.7759/cureus.113228

**Published:** 2026-07-23

**Authors:** Jessica Colon, Anna Falabella

**Affiliations:** 1 Graduate Medical Education, Memorial Healthcare System, Pembroke Pines, USA; 2 Dermatology, Hollywood Dermatology, Miramar, USA

**Keywords:** atopic dermatitis, biologic therapy, drug induced vitiligo, dupilumab, nemolizumab, repigmentation, ruxolitinib, tacrolimus, vitiligo

## Abstract

Dupilumab, a monoclonal antibody targeting the IL-4 and IL-13 pathways, has revolutionized the treatment of moderate-to-severe atopic dermatitis (AD). However, emerging evidence suggests rare immune-mediated adverse events, including vitiligo onset or exacerbation. The pathogenesis involves potential immune dysregulation and cytokine imbalance following IL-4/IL-13 inhibition. To report a case of dupilumab-induced vitiligo in a patient with AD and evaluate the therapeutic alternatives for management, we present a case report of a 57-year-old woman with longstanding AD who developed new-onset vitiligo following dupilumab therapy. The patient was subsequently switched to nemolizumab, an IL-31 receptor antagonist, and treated with topical tacrolimus 0.1% ointment on one hand and ruxolitinib cream on the contralateral hand to compare treatment efficacy. Three months after dupilumab initiation, the patient developed depigmented patches on bilateral metacarpophalangeal joints consistent with non-segmental vitiligo, with no prior history of vitiligo or autoimmune disease. Following transition to nemolizumab and topical treatments, significant improvement occurred within eight weeks. The hand treated with ruxolitinib demonstrated superior repigmentation compared to the tacrolimus-treated hand. This case highlights dupilumab’s potential to induce vitiligo through Th2 cytokine inhibition and subsequent Th1/Th17 activation, promoting melanocyte destruction. The superior efficacy of topical JAK-STAT inhibition with ruxolitinib compared to tacrolimus suggests JAK pathway modulation may be more effective for managing biologic-induced pigmentary disorders. Clinicians should monitor for the development of vitiligo in dupilumab-treated patients.

## Introduction

Atopic dermatitis (AD) is a chronic, relapsing inflammatory skin disease characterized by pruritus and eczematous lesions, often significantly impacting quality of life [1]. In recent years, biologic therapies have revolutionized the treatment of moderate-to-severe AD, with dupilumab emerging as the first monoclonal antibody targeting IL-4 and IL-13 pathways [2]. Since its approval, dupilumab has demonstrated remarkable efficacy in clinical trials and real-world settings, offering significant relief for patients with treatment-resistant disease.

Despite its favorable profile, emerging evidence points to rare but notable immune-mediated adverse events, including the onset or exacerbation of vitiligo, an acquired depigmenting disorder resulting from melanocyte loss [3-6]. To date, only a small number of cases of dupilumab-associated vitiligo have been reported in the literature, largely as isolated case reports and small case series. However, a recent scoping review by Mehta et al. found a mean latency of approximately three months between dupilumab initiation and vitiligo onset across the reported cases, suggesting a potential temporal signal despite the limited overall number of cases [7]. The pathogenesis of vitiligo in the context of biologic therapy remains incompletely understood but is hypothesized to involve immune dysregulation and cytokine imbalance, particularly a proposed shift from Th2-predominant signaling toward Th1 and Th17 responses following IL-4/IL-13 blockade, which may promote melanocyte destruction [3]. As more patients with AD are treated with targeted biologics, recognizing and managing such complications becomes increasingly important, and continued case-level reporting remains an important tool for characterizing this uncommon but clinically significant adverse effect.

The present case report describes a woman with longstanding AD who developed new-onset vitiligo following dupilumab therapy, with subsequent improvement after switching to nemolizumab, an IL-31 receptor antagonist, and topical tacrolimus and ruxolitinib use. This case contributes to the growing literature on biologic-induced pigmentary disorders and provides novel insights into potential therapeutic alternatives for affected patients, while highlighting important limitations that should inform interpretation of these observations.

## Case presentation

**Figure 1:**
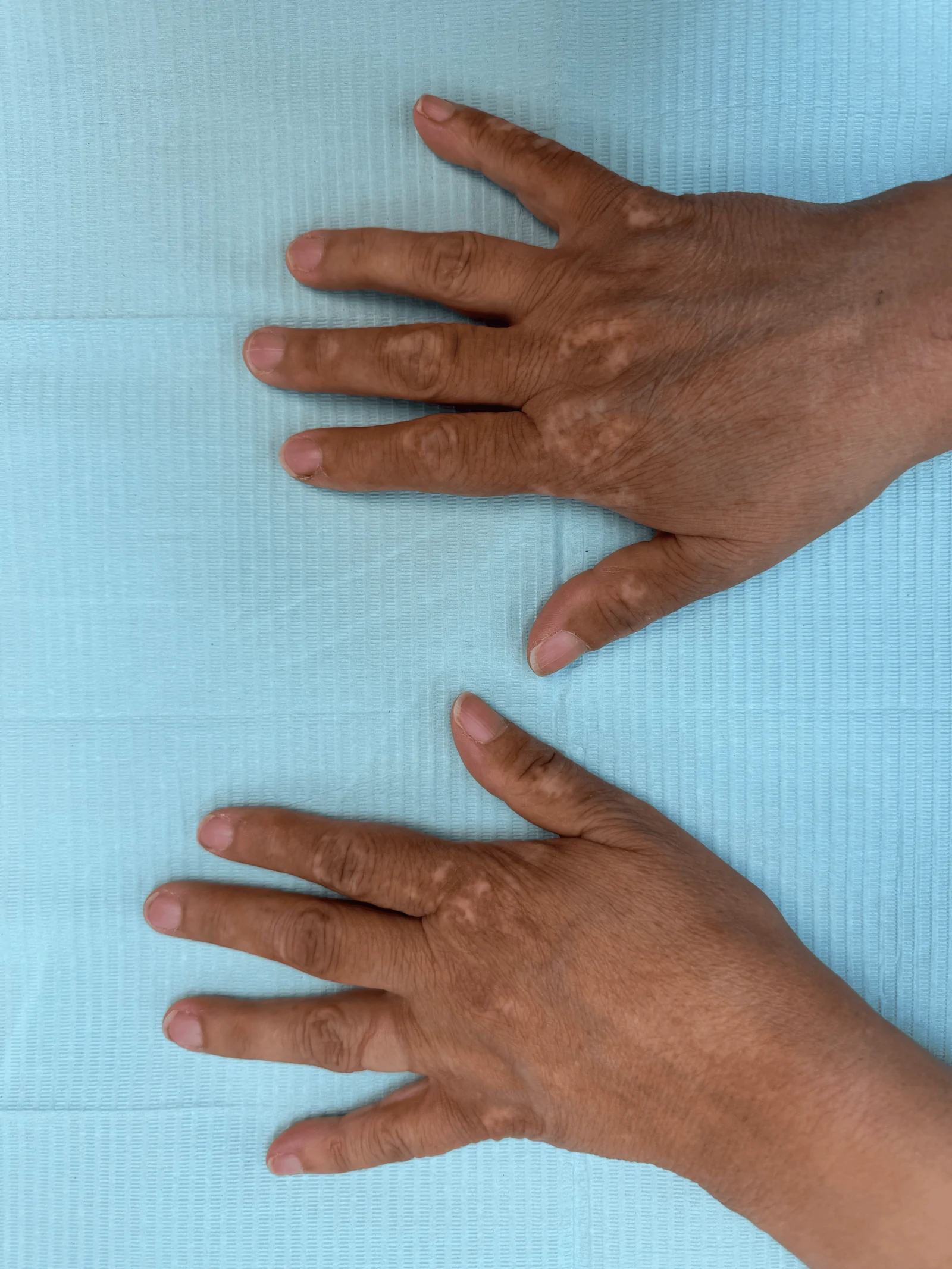
Bilateral hand vitiligo that developed three months after dupilumab initiation, showing characteristic depigmented patches on the dorsal aspect of both hands. Baseline involvement was more extensive on the right hand than the left hand.

A 57-year-old woman with a history of eczema as a child initially presented with ill-defined erythematous scaly plaques on the body, consistent with a diagnosis of atopic dermatitis. Due to the large percentage of body surface area affected, she was initiated on dupilumab, with a loading dose subcutaneous injection of 600 mg on day zero, followed by a 300 mg subcutaneous injection every two weeks. At her three-week follow-up appointment, the patient reported diminished efficacy of dupilumab, as she continued to experience full-body flares. Her dermatologist prescribed mometasone 0.1% cream, hydrocortisone 2.5% cream, and Epiceram to use to further manage her flares, in conjunction with dupilumab injections. Three months later, the patient presented with depigmented patches on the left and right metacarpophalangeal joints, consistent with a diagnosis of non-segmental vitiligo (Figure 1). The patient was Fitzpatrick skin type IV. Diagnosis was confirmed clinically with Wood's lamp examination, which demonstrated enhanced fluorescence of the depigmented patches, and dermoscopy, which showed features typical of vitiligo, including a residual perifollicular pigment pattern. Histopathologic confirmation via skin biopsy was not performed, as the clinical and dermoscopic findings were considered sufficiently characteristic of vitiligo. No autoimmune serologic workup, including thyroid function testing or autoantibody panels, was performed, and the patient reported no family history of vitiligo or other autoimmune disease.

**Figure 2:**
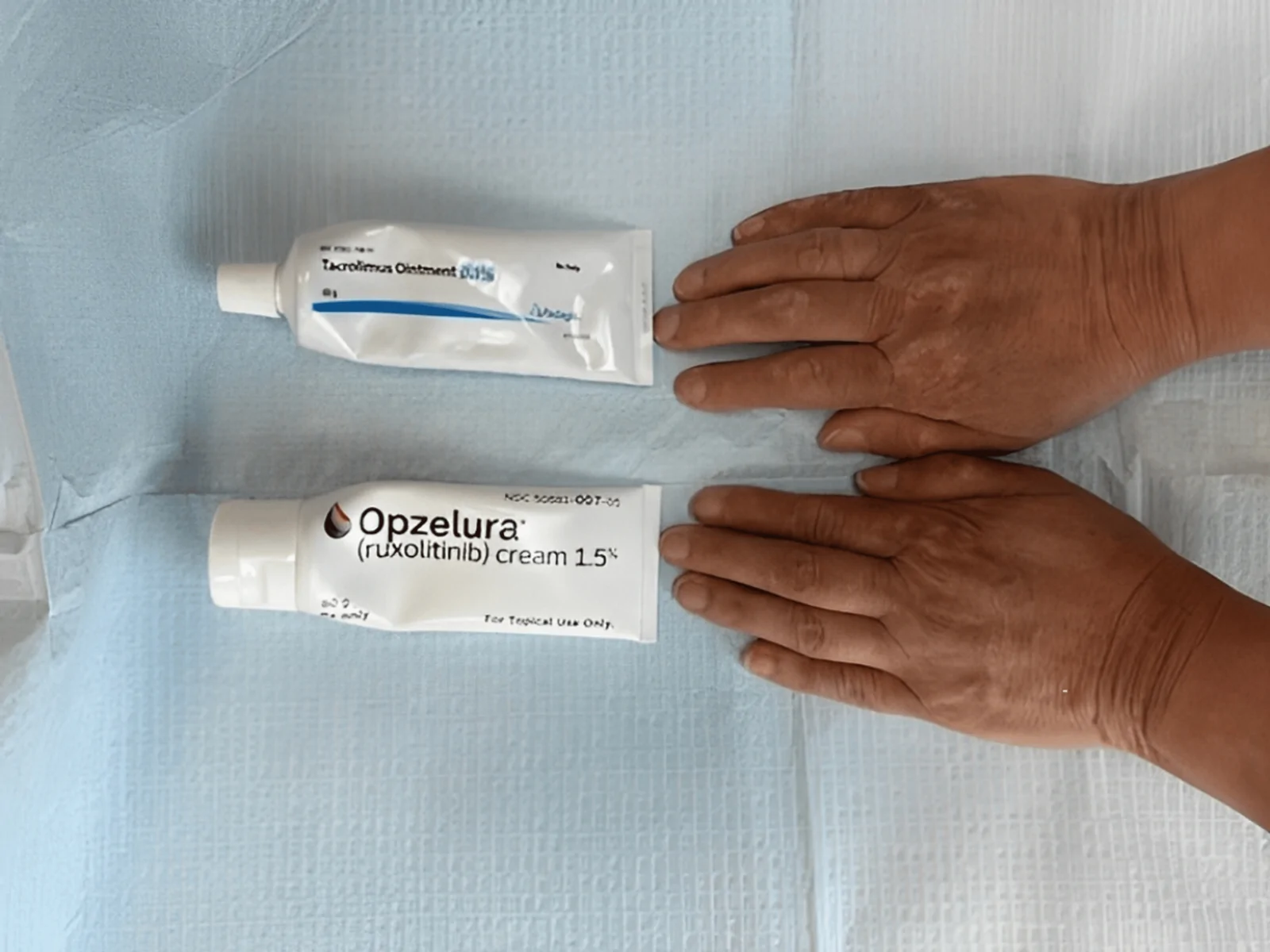
Clinical improvement at two months after dupilumab discontinuation and initiation of targeted therapies The left hand received topical tacrolimus 0.1% ointment; the right hand received topical ruxolitinib cream. Both hands were treated concurrently with systemic nemolizumab. Repigmentation is visually more pronounced on the ruxolitinib-treated right hand, though this hand also had greater baseline involvement (Figure 1).

The patient denied any prior history of vitiligo or autoimmune disease, and this was her first exposure to an injectable biologic medication. She was prescribed tacrolimus 0.1% ointment and ruxolitinib in an effort to reverse the vitiligo and was instructed to apply tacrolimus to one hand and ruxolitinib to the other hand, allowing for a side-by-side comparison of treatment efficacy (Figure 2).

Following the emergence of vitiligo-like depigmentation suspected to be associated with dupilumab use, the patient was transitioned to nemolizumab. Within eight weeks of switching biologic medications and maintaining consistent use of ruxolitinib and tacrolimus, the patient experienced significant improvement in her vitiligo, with more repigmentation noted on the hand treated with ruxolitinib.

## Discussion

This case highlights two important observations: first, that dupilumab therapy was temporally associated with the onset of vitiligo in a patient with no prior personal or family history of the condition, and second, that topical ruxolitinib was associated with more pronounced repigmentation than tacrolimus in this patient, an observation that warrants further controlled study given important limitations discussed below.

The association between dupilumab therapy and new-onset vitiligo is increasingly recognized in the literature, though this relationship remains largely inferred from temporal proximity rather than confirmed through mechanistic or laboratory evidence [3-6]. Several case reports have documented vitiligo development within months of treatment initiation, and some have described rapid enlargement of pre-existing vitiligo lesions following dupilumab initiation, suggesting the drug may exacerbate existing subclinical disease [4]. A literature survey conducted by Mehta et al. reported an average latency of 3.11 months from dupilumab initiation to vitiligo onset, which aligns with the timeline observed in our patient [7]. The incidence of dupilumab-induced vitiligo appears to be low but may be underreported due to limited clinician awareness of this adverse effect. In this case, no autoimmune serologic workup, including thyroid function testing or autoantibody panels, was performed, and the patient reported no family history of vitiligo or other autoimmune disease. Because this evaluation was not pursued, alternative or contributing causes, such as subclinical autoimmune thyroid disease or an independent, coincidentally timed autoimmune process, cannot be excluded. The temporal relationship observed here is consistent with prior reports but should be interpreted as a plausible association rather than established causation.

The development of vitiligo during dupilumab therapy has been proposed to involve complex immunological mechanisms related to the drug's effects on cytokine balance. One hypothesis suggests that inhibition of Th2 cytokines (IL-4 and IL-13) by dupilumab may shift the immune balance toward Th1 and Th17 responses, which are implicated in mediating melanocyte destruction in vitiligo [3]. This shift could theoretically unmask latent autoimmune processes or trigger de novo autoimmunity against melanocytes, though this mechanism has not been directly confirmed in our patient. IL-13 also plays a role in maintaining skin barrier function and regulating local inflammatory responses [8], and blockade of IL-4 signaling may alter the cutaneous microenvironment in ways that could indirectly affect melanocyte survival through changes in keratinocyte-melanocyte interactions or local immune surveillance [9]. These mechanisms remain hypothetical in the context of this case and are drawn from the broader literature rather than from patient-specific mechanistic data.

This case also provided an opportunity to observe two topical vitiligo treatments applied concurrently, as tacrolimus 0.1% ointment was applied to the left hand while ruxolitinib cream was applied to the right hand, with both hands treated alongside systemic nemolizumab. Over the eight-week observation period, repigmentation appeared more pronounced on the ruxolitinib-treated hand. However, this observation carries several important limitations. Baseline disease severity differed between the two hands, with more extensive depigmentation present on the right hand prior to treatment initiation, which may itself influence the apparent degree of visible improvement independent of treatment effect. Additionally, this comparison is based on a single patient, relied on visual assessment rather than objective or quantitative outcome measures, and involved simultaneous discontinuation of dupilumab, initiation of nemolizumab, and introduction of both topical therapies. As a result, this case cannot isolate which intervention, or combination of interventions, was primarily responsible for the observed clinical improvement, and the finding should not be interpreted as evidence that ruxolitinib is more effective than tacrolimus. It is reported descriptively as an observed clinical outcome that may help generate hypotheses for future controlled comparison. Prior literature has proposed that the JAK-STAT pathway plays a role in vitiligo pathogenesis, with JAK inhibitors showing promise in modulating interferon-gamma signaling and other inflammatory cascades involved in vitiligo progression [10], which may offer biologic plausibility for the pattern observed here, though it does not substitute for a controlled comparison.

## Conclusions

This case underscores the importance of clinical awareness regarding vitiligo as a potential adverse effect of dupilumab therapy in patients with atopic dermatitis. Recognizing this complication early allows clinicians to reconsider biologic selection and pursue targeted topical treatment promptly, which may improve outcomes for affected patients. Our experience raises the hypothesis that topical JAK-STAT inhibition may offer a repigmentation benefit over calcineurin inhibition in this setting, though this observation is limited by the single-patient design and baseline asymmetry between treated sites, and should not be interpreted as demonstrating therapeutic superiority. Controlled, prospective studies with objective outcome measures are needed to evaluate this possibility. Continued clinician vigilance, prompt recognition, and individualized treatment planning remain essential when managing dupilumab-associated vitiligo. Larger, prospective studies are needed to validate these therapeutic approaches and to further clarify the underlying immunologic mechanisms driving this adverse effect, ultimately helping guide optimized, patient-centered care for individuals receiving biologic therapy for atopic dermatitis.
